# High-throughput detection of antioxidants in mulberry fruit using correlations between high-resolution mass and activity profiles of chromatographic fractions

**DOI:** 10.1186/s13007-017-0258-3

**Published:** 2017-12-06

**Authors:** Ye Ji Park, Si Hyun Seong, Min Sun Kim, Sang Wan Seo, Mee Ree Kim, Hyun Sik Kim

**Affiliations:** 1 Mass Spectrometry and Advanced Instrumentation Group, Korea Basic Science Institute, Cheongju, Chungcheongbuk-do 28119 Korea; 2 College of Human Ecology, Chungnam National University, Daejeon, 34134 Korea; 3 College of Pharmacy, Chungnam National University, Daejeon, 34134 Korea; 4 Department of Oriental Medicine and Biotechnology, Honam University, Gwangju, 62399 Korea

**Keywords:** *Morus alba*, FT-ICR MS, Isotopic fine structure, Molecular formula determination, SCAMP, DPPH, Scoring, Enrichment, AUC, Discovery, Functional molecule

## Abstract

**Background:**

Plant extracts contain a huge variety of pharmacologically active substances. Conventionally, various chromatographic methods must be applied several times to purify functional compounds to measure their functional activity. However, conventional purification methods are time-consuming and expensive due to the laborious purification process. Recently, a high-throughput discovery method that replaces such time-consuming purification processes was introduced; this method uses 15 T ultra-high-resolution Fourier transform ion cyclotron resonance mass spectrometry (15 T FT-ICR MS) and a high-throughput screening method. This 15 T FT-ICR MS provides unparalleled resolution and sub-ppm accuracy in mass measurements, while simultaneously detecting multiple compounds without separation. The high-throughput, simultaneous multi-component discovery method known as Scaling of Correlations between Activity and Mass Profiles (SCAMP) was used to detect functional compounds in a plant extract. We validated the performance of SCAMP using 33 fractions from antioxidant-rich mulberry ethyl acetate extract and known standard antioxidants.

**Results:**

The mulberry fruit was first separated into 33 fractions by LC and analyzed using high-resolution mass spectrometry. The antioxidative strength of the 33 fractions and standard antioxidants was measured. To validate the efficiency of this antioxidant discovery method, correlations between the antioxidation activity profile and changes in mass intensity of components within the 33 fractions were calculated to provide relative scores for the antioxidant candidate list. Enrichment curves and area under the curve (AUC) values were then calculated to compare the performance of the methods. Using this improved scoring method, five strong antioxidants, chlorogenic acid (14.2 ng), dihydoxy quercetin (46.2 ng), rutin (154.0 ng), quercetin (71.7 ng) and luteolin (3.5 ng) in 2 kg mulberry fruit, were found within the top 20 candidates.

**Conclusions:**

We calculated AUCs in order to compare scoring methods quantitatively. Scoring systems were compared and calculated AUCs, where the AUCs for new scoring systems (0.98 and 0.99) were higher than the previously used correlation coefficient (AUC = 0.89). Using the new scoring algorithms, we successfully enriched thirteen unknown strong antioxidant candidates in addition to known antioxidants, methyl syringin and naringenin (3.5 ng) in mulberry extract. Targeted purification of these unknown candidates will significantly reduce purification time and labor.

**Electronic supplementary material:**

The online version of this article (10.1186/s13007-017-0258-3) contains supplementary material, which is available to authorized users.

## Background

**Fig. 1:**
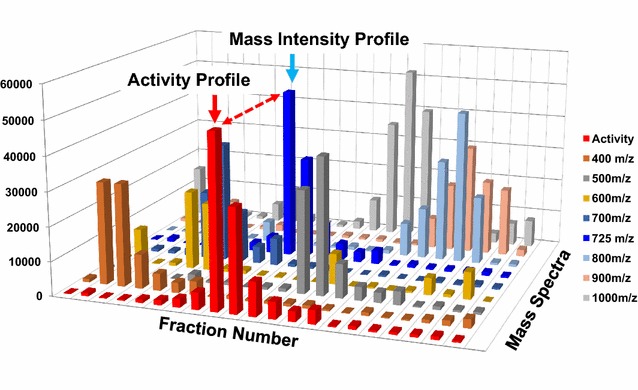
A conceptual representation of the Scaling of Correlations between Activity and Mass Profiles (SCAMP) method. If the relative activity of sequentially separated chromatographic fractions can be measured and the mass intensity for a given compound shows a similar trend to that of the total activity change over all fractions, then that compound may contribute to the total activity

Plant extracts contain a huge number of pharmacologically active substances [1–3]. Conventionally, various preparative chromatographic methods are applied several times to purify functional compounds in order to estimate their functional activity. However, conventional purification methods [4] are time-consuming, complex, and expensive due to the purification process necessary prior to activity assays of functional components. Recently, a high-throughput multi-component discovery method that does not need time-consuming purification steps was introduced [5]; this method combines 15 T ultra-high-resolution Fourier transform ion cyclotron resonance mass spectrometry (15 T FT-ICR MS) and high-throughput screening. Ultra-high-resolution Fourier transform ion cyclotron resonance mass spectrometry (UHR FT-ICR MS) provides unparalleled resolution and sub-ppm accuracy in mass measurements [6–8], while simultaneously detecting multiple ions in a mixture without separation. UHR FT-ICR MS can be used to determine the molecular formula of small organic molecules (less than 500 amu) if the molecular mass is measured at 1 ppm accuracy alongside its isotopic pattern [9]. UHR FT-ICR MS can be used to determine isotopic fine structure (IFS) and thus the molecular formula for small organic compounds [10, 11]. If the relative activity of sequentially separated chromatographic fractions can be measured with high-throughput screening, and if the mass intensity for a given compound shows a similar trend to that of the total activity change over all fractions (as shown in Fig. 1), then that compound may contribute to the total activity of the fraction. A high-throughput simultaneous multi-component discovery method, known as Scaling of Correlations between Activity and Mass Profiles (SCAMP), was proposed to discover functional compounds in plant extracts; this method uses the correlation between mass intensity profiles and activity profiles of sequential fractions [5]. If the relative correlation coefficient between mass intensities and activity profiles can be calculated, active candidates and their relative activities can be quickly identified. However, preliminary results from early stages of SCAMP have included only a few fractions (specifically, 11 fractions) to show correlation behavior statistically [5]. Thus, we measured antioxidation activity and acquired UHR mass spectra using 33 fractions from antioxidant-rich mulberry ethyl acetate extract (EAEM), as well as known standard antioxidant solutions (SAOx). All the standard antioxidants are selected from the published antioxidant list [12–18]. SCAMP performance was validated using UHR MS and activity data by listing strong antioxidant candidates. SAOx will likely be listed within the high score region due to high correlation scores between the ultra-high-resolution mass peak intensity profile of each component and the antioxidation activity profile from the 33 chromatographically separated fractions. Certain chemicals showed chromatographic distributions within several fractions, which is similar to activity change behavior. An accurate active compound mass can then be determined using ultra-high-resolution 15 T FT-ICR electrospray ionization mass spectrometry (UHR ESI MS), and the exact chemical formulae can be determined directly using accurate mass values and the isotopic fine structure from mass peaks [10]. Therefore, a probable candidate list with their chemical formulae can be quickly determined. Here, this list of possible antioxidants and their chemical formula from mulberry extracts were annotated by searching natural product databases to validate the updated performance of our new scoring algorithm adopted within SCAMP. In addition, two new scoring algorithms were suggested to improve the efficiency of active compound enrichment. The first method considers the summed peak intensity value, in addition to the behavioral similarity index (correlation coefficient), to account for the concentration dependence of activity. The second method resolves different components within the same mass peak by identifying that peak in multiple fractions. Therefore, certain mass peaks appear discontinuously through sequential fractions due to their different chromatographic retention properties. If certain components have different structures but the same mass, then those components will be separated chromatographically, with different retention times, and will elute into different fractions. This grouping strategy enhances the specificity of functional compound discovery using SCAMP by avoiding overestimation of correlation coefficients by using additional scores for multiple components in the same mass peak.

## Methods

All MS was performed using both 15 T FT-ICR (SolariX; Bruker Daltonics, Billerica, MA, USA) and Q-TOF (SYNAPT G2-Si High Definition Mass Spectrometer; Waters Co., Milford, MA, USA) mass spectrometers. UHR ESI/FT-ICR MS was used to profile compounds in fractions and determine the molecular formulae of compounds; UPLC/MS was employed to identify SAOx in mulberry extracts using high-resolution mass profiling and MSMS.

### Sample preparation

**Fig. 2:**
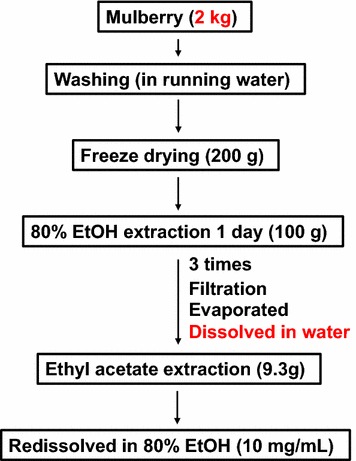
Sample preparation. Dried ground mulberry fruit was extracted using 80% ethanol over 3 days and then extracted using ethyl acetate to yield the ethyl acetate fraction

All standards were purchased from Chengdu Must Bio-technology and Wuhan ChemFaces Biochemical (China). Methanol (HPLC-grade), ethanol, acetonitrile, and ethyl acetate were purchased from J.T. Baker (Phillipsburg, NJ, USA). Dried fruits of *Morus alba* were purchased from a local herbal market in Gyeongbuk, South Korea. Fruit samples were stored at – 20 °C immediately after collection. Fresh fruit samples (2 kg) of *Morus alba* were lyophilized at − 40 °C (FD 8518; Ilshin Lab. Co., Ltd., Yangju, South Korea). After drying, the samples were ground and sieved through a 0.3-µm mesh, and stored at − 20 °C until analysis. Dried mulberry fruit (200 g) was extracted three times using 1 L of 80% ethanol over 3 days at room temperature as shown in Fig. 2. This 80% ethanol extract was evaporated and then suspended in 1000 mL of distilled water and successively partitioned three times with the same volume of ethyl acetate to yield the ethyl acetate fraction (9.3 g).

### UPLC/MS and MS/MS

Samples (5 μL) were prepared by diluting the ethyl acetate extract from mulberry (EAEM) to 10 mg/mL with methanol. Samples were then analyzed by UPLC/Q-TOF MS. UPLC/MS analysis was performed on a Waters ACQUITY UPLC system coupled with a Waters SYNAPT G2-Si High Definition Mass Spectrometer equipped with an electrospray ionization (ESI) source (Waters Co.). Chromatographic separation was carried out using an ACQUITY UPLC HSS C18 (100 mm × 2.1 mm, 1.8 μm) column at 40 °C, and an auto-sampler temperature of 10 °C. Mobile phase A consisted of water, while mobile phase B consisted of acetonitrile. The solvent gradient was initially 3% B, changing to 3%–29% B over 50 min at a flow rate of 0.3 mL/min. This was followed by a 15-minute washing period using 95% B prior to the next sample injection. Chromatograms were monitored at 310 nm using a UV detector. For the UPLC-HDMS analysis, capillary voltage and cone voltage were set at 2.0 kV in negative ion electrospray mode. Other conditions included a source temperature of 100 °C, desolvation gas flow of 900 L/h at a temperature of 500 °C, an extraction cone voltage of 4.0 V, a collision energy of 4 eV, and a cone gas flow of 0 L/h. The scan range was m/z 50–1500. Mass scale was corrected during acquisition using an external reference (Lock-Spray), which consisted of a 300 ng/mL solution of leucine enkephalin (Sigma Aldrich) at a flow rate of 20 μL/min; this generated a reference ion in negative ion mode ([M-H]^−^ with m/z = 554.2615) to ensure accuracy and reproducibility. All data were acquired using MassLynx™ (V4.2) software in centroid mode.

### Sample fractionation (Prep LC)

Analysis was carried out using an HPLC system (Shimadzu, Japan) equipped with an LC-20AR pump, SPD-20A detector, SIL-10AP auto-sampler, and an FRC-10A fraction calculator. Each of three different amounts (300, 540, and 1080 mg) of EAEM were diluted with 5 mL of ethanol and injected onto a C18 column (250 × 21.2 mm, 7 μm, PrepHT 300SB; Agilent, Santa Clara, CA, USA), respectively, and separated using a binary mobile phase composed of solvents A (water) and B (acetonitrile) at a flow rate of 10.0 mL/min. During this, a solvent gradient of 3% B for 0–5 min, 3%–29% B for 5–50 min, and 29%–56% B for 50–65 min was used. This was followed by a 15-min equilibrium period under initial conditions prior to sample injection. Chromatograms were monitored at 310 nm using the UV detector. Fractions were collected every 2 min and up to 33 fractions were collected in total. After fractionation, three sets of 33 fractions were merged into a single set of 33 fractions.

### UHR 15 T FT-ICR MS

Mass profiling was performed using a 15 T FT-ICR mass spectrometer (SolariX system; Bruker Daltonics, Bremen, Germany) in negative ESI mode. Stock solutions of all 33 fractions were diluted 1000-fold with 100% methanol and introduced into the FT-ICR mass spectrometer using direct infusion (Triversa NanoMate; Advion BioSciences, Ithaca, NY, USA) at a flow rate of 400 nl/min. MS operating parameters included a capillary voltage of 2.0 V, a mass range (m/z) from 122 to 1200, an accumulation time of 0.7 s, a scan number of 100, and a sine bell apodization window function applied to the time-domain signal. External calibration was performed via quadratic regression using a 0.1 mg/mL arginine solution. All data were processed using DataAnalysis (V.4.4; Bruker Daltonics). Isotopic masses and abundances used for theoretical mass calculations were provided by the National Institute of Standards and Technology [19].

### Determination of DPPH radical-scavenging activity

The DPPH radical-scavenging assay was performed in accordance with a previously described method [20], with all tests being performed in triplicate. Ascorbic acid was used as a positive control. Antioxidation activity profiles were generated by measuring the radical-scavenging activity of each fraction using 1,1-diphenyl-2-picrylhydrazyl (DPPH). Each fraction was dried with a centrifuge using a vacuum concentrator (Hanil Science Industrial, Incheon, South Korea) and reconstituted in methanol to make a 10 mg/mL stock solution. All 33 stock solutions were diluted with 50% ethanol and mixed vigorously with 100 µL of 300 µM aqueous DPPH solution. The absorbance of the remaining DPPH was measured at 518 nm after 30 min as follows:

$$ \%_{\text{DPPH}} = \left( {\frac{{{\text{Ab}}_{\text{t}} - {\text{Ab}}_{\text{b}} }}{{{\text{Ab}}_{0} }}} \right) \times 100\% $$

In this equation, Ab_t_ = absorbance of the DPPH solution with tested extracts; Ab_0_ = the absorbance of the DPPH solution upon addition of ethanol/water (1:1, v/v); and Ab_b_ = absorbance of tested extract solutions with the addition of 95% ethanol.

## Results and discussion

### UPLC/MS and MS/MS

**Fig. 3:**
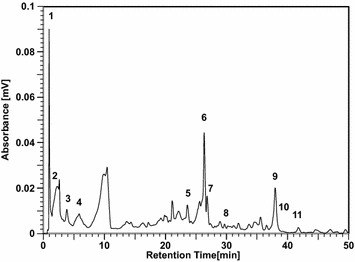
UPLC/MS chromatogram of ethyl acetate extract of mulberry fruit. Ethyl acetate extracts from mulberry (EAEM) were analyzed by UPLC/Q-TOF MS and eleven SAOx were clearly detected from EAEM: (1) gallic acid, (2) gentisic acid, (3) chlorogenic acid, (4) caffeic acid, (5) dihydroquercetin, (6) rutin, (7) quercetin-3-o-glucoside, (8) quercitrin, (9) quercetin, (10) luteolin, (11) kaempferol. Each standard anti-oxidant in the EAEM was identified using the mass values of fragment and parent ions identified by LC/MS, and LC/MSMS analyses of pure SAOx and EAEM

**Fig. 4:**
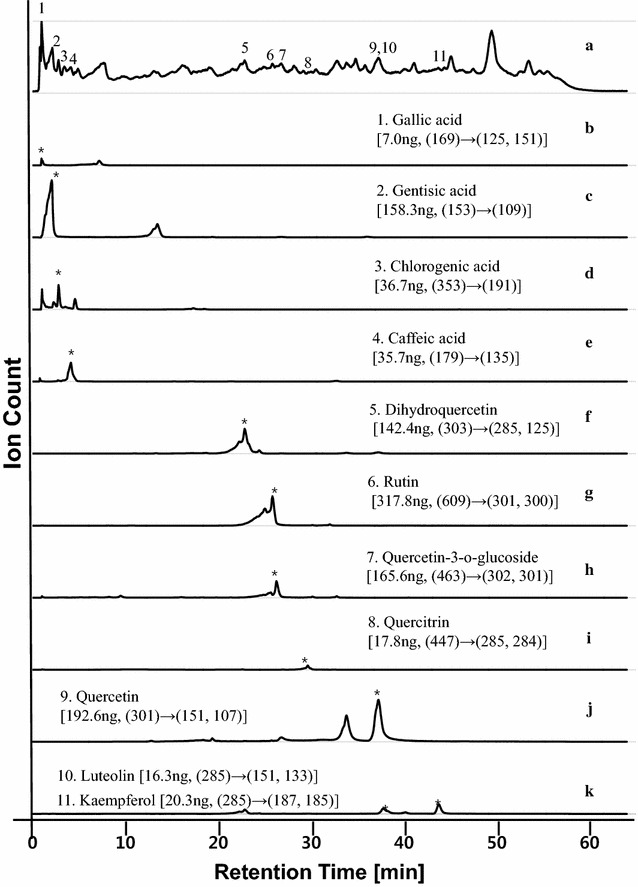
Total ion chromatogram and selected ion chromatogram of EAEM.Ethyl acetate extracts from mulberry (EAEM) were analyzed by UPLC/Q-TOF MS and MSMS and eleven SAOx were clearly detected from EAEM. (a) Total ion chromatogram of EAEM. Selected ion chromatograms of SAOx were shown in (b) gallic acid, (c) gentisic acid, (d) chlorogenic acid, (e) caffeic acid, (f) dihydroquercetin, (g) rutin, (h) quercetin-3-o-glucoside, (i) quercitrin, (j) quercetin, and (k) luteolin and kaempferol. Each standard anti-oxidant in the EAEM was identified using the mass values of parent and fragment ions by LC/MS and LC/MSMS of pure SAOx and EAEM

**Fig. 5:**
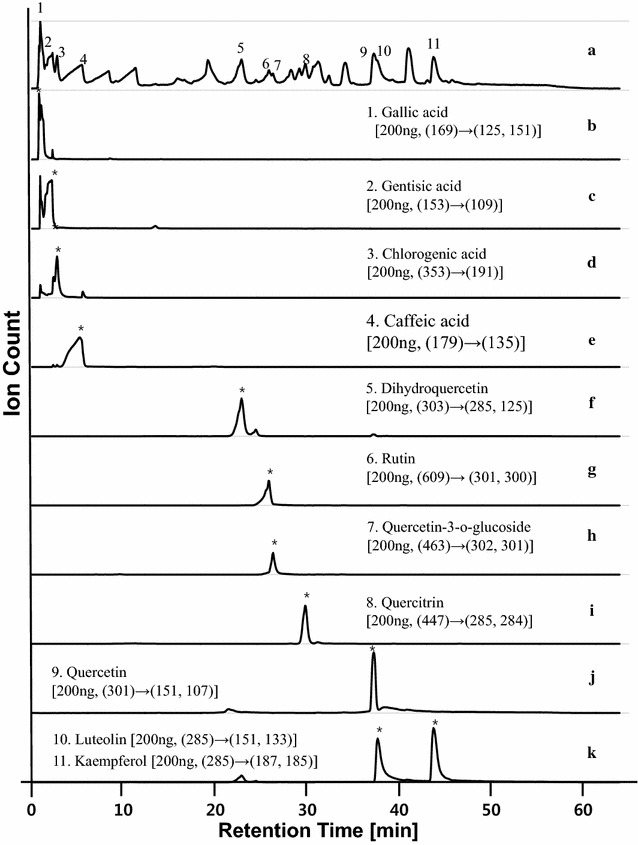
Total ion chromatogram and selected ion chromatogram of standard antioxidant mixture. A standard antioxidant mixture was analyzed by UPLC/Q-TOF MS and eleven SAOx were clearly detected from the SAOx mixture : (a) total ion chromatogram, (b) gallic acid, (c) gentisic acid, (d) chlorogenic acid, (e) caffeic acid, (f) dihydroquercetin, (g) rutin, (h) quercetin-3-o-glucoside, (i) quercitrin, (j) quercetin, and (k) luteolin and kaempferol. Each standard anti-oxidant in the SAOx mixture was identified using the mass values of fragment and parent ions identified by LC/MS, and LC/MSMS analyses of pure SAOx mixture

UPLC/MS of the extracts was performed to confirm that the contents of standard antioxidants (SAOx) were sufficient to produce mass profiles within all 33 fractions. In LC/MS and MSMS experiments, 11 SAOx were clearly detected from EAEM (as shown in Figs. 3 and 4). Each standard anti-oxidant in the EAEM was identified using the mass values of fragment and parent ions identified by LC/MS, and LC/MSMS analyses of pure SAOx and EAEM as shown in Figs. 4 and 5. Quantification was performed by examination of UV absorption chromatograms and selected ion chromatograms of SAOx. The amount of SAOx used within the EAEM indicates that the sensitivity of this method is ~ 3 ng; this means that if certain antioxidants were included in the EAEM below ~ 3 ng, we may not detect them using current SCAMP experimental conditions. Improving the sensitivity of mass spectrometry and activity assay systems could improve detection sensitivity, but the necessary accumulation of data required for improving the signal-to-noise ratio (S/N) of mass peaks of components would be time-consuming. Table 1 shows the approximate contents, DPPH anti-oxidation EC_50_, MS/MS fragment ion list, retention time, and high-resolution mass and chemical formula of SAOx; however, the low nanogram sample used to provide the content change through the sequential fractions from preparative LC of EAEM was barely detected in our 15 T FT-ICR MS experiment. Determining antioxidation activity is critical for estimating the performance of SCAMP. Thus, the DPPH radical-scavenging activity of all 11 SAOx was measured. Each fraction was analyzed with UPLC/MS and MSMS to determine the chromatographic behavior of each SAOx within certain fraction regions. Thus, although UHR FT-ICR MS only produces fraction mass profiles, it is still possible to distinguish between components with identical masses but different structures. This is because the mass distribution through sequentially separated fractions shows different chromatographic behaviors and has unique distributions.

**Table 1:** UPLC/MSMS identification of standard anti-oxidant compounds in the EtOAc extract of mulberry fruit

	Standard name	Formula	Calc. [M-H]^−^ [m/z]	Observed [M-H]^−^ [m/z]	Error [mDa]	Retent ion time	MS/MS fragment ion peaks (relative intensity %)	EC_50_ [uM]	Contents [ng]
1	Gallic acid	C_7_H_6_O_5_	169.0143	169.0144	+ 0.1	0.94	151.00(33), 125.02(100)	7.8	7.0
2	Gentisic acid	C_7_H_6_O_4_	153.0193	153.0184	− 0.9	2.05	109.02(100)	47.2	158.3
3	Chlorogenic acid	C_16_H_18_O_9_	353.0878	353.0873	− 0.5	2.79	191.05(100)	23.6	36.7
4	Caffeic acid	C_9_H_8_O_4_	179.0350	179.0357	+ 0.7	4.12	135.04(100)	38.8	35.7
5	Dihydro -quercetin	C_15_H_12_O_7_	303.0510	303.0557	+ 4.7	22.83	285.04(100), 259.06(6), 177.02(18), 125.02(20)	14.0	142.4
6	Rutin	C_27_H_30_O_16_	609.1461	609.1536	+ 7.5	25.83	301.03(75), 300.02(100), 302.03(14)	10.7	317.8
7	Quercetin-3-o-glucoside	C_21_H_20_O_12_	463.0882	463.0953	+ 7.1	26.25	302.03(18), 301.03(100), 257.04(13),	29.0	165.6
8	Quercitrin	C_21_H_20_O_11_	447.0933	447.0939	+ 0.6	29.62	287.05(53), 285.04(53.83), 284.03(72), 151.00(27),	20.7	17.8
9	Quercetin	C_15_H_10_O_7_	301.0354	301.0337	− 1.7	37.20	175.04(19), 151.00(32), 149.02(10), 107.01(13)	13.9	192.6
10	Luteolin	C_15_H_10_O_6_	285.0405	285.0424	+ 1.9	37.76	199.04(13), 151.00(32), 149.02(10), 134.03(10), 133.02(100), 107.01(13)	11.9	16.3
11	Kaempferol	C_15_H_10_O_6_	285.0405	285.0424	+ 1.9	43.70	239.03(17), 229.05(16), 227.03(11), 211.04(15), 187.04(18), 185.06(20), 171.04(11), 159.04(15), 143.05(11)	30.1	20.3

The approximate contents, DPPH anti-oxidation EC_50_, MS/MS fragment ion list, retention time, and high-resolution mass and chemical formula of SAOx were listed for the EtOAc extract of mulberry fruit

## Sample fractionation (Prep LC)

**Fig. 6:**
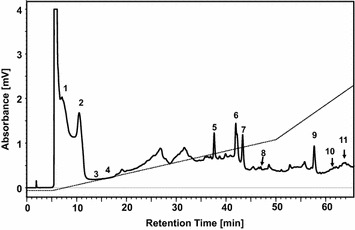
Preparative HPLC chromatogram of *Morus alba L.* EtOAc extract. Ethyl acetate extracts (EAEM) from 80% ethanol extracts of mulberry fruit were analyzed using preparative liquid chromatography and all SAOx were detected at similar retention times with annotated UPLC chromatograms as annotated with the same number inside the chromatogram

Ethyl acetate extracts (EAEM) from 80% ethanol extracts of mulberry fruit were analyzed using preparative liquid chromatography (Fig. 6). To find active compounds that exhibit behavior similar to activity profiles within sequential fractions, 33 fractions were collected sequentially every 2 min. All SAOx were detected at similar retention times with annotated UPLC chromatograms as shown in Figs. 3 and 6. Using UPLC/MS of the fractions separated by Prep LC and the selected ion chromatogram of SAOx, we observed the large amounts of dihydroquercetin, rutin and quercetin-3-o-glucoside with trace amounts of luteolin and kaempferol as shown in Figs. 5 and 6.

**Fig. 7:**
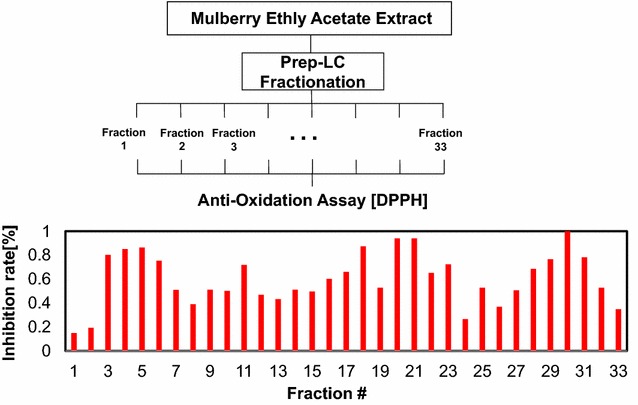
Fractionation and Activity data generation. Ethyl acetate extracts (EAEM) from mulberry fruit were analyzed using preparative liquid chromatography and sequential fractions, 33 fractions were collected sequentially every 2 min. The antioxidation activity profile of each fraction was generated by measuring the radical scavenging activity of all 33 fractions using 1,1-diphenyl-2-picrylhydrazyl (DPPH)

The antioxidation activity profile of each fraction was generated by measuring the radical scavenging activity of all 33 fractions using 1,1-diphenyl-2-picrylhydrazyl (DPPH). The relative inhibition rates are plotted in Fig. 7. The relative antioxidation strength was then normalized by scavenging activity (Additional file 1: Table S1). SCAMP calculations were used to discover antioxidant compounds by combining all 33 mass spectra and activity data from the fractions. SCAMP performance was validated and improved by analyzing SAOx (previously reported [12–18] as anti-oxidative constituents of mulberry extract) by LC/MS. The antioxidant’s radical scavenging activity was then measured using 1,1-diphenyl-2-picrylhydrazyl (DPPH) assays to provide EC_50_ values for all of the SAOx, given in Table 1. Eleven SAOx were observed in EAME using LC/MS and LC/MSMS, where the performance of scoring systems of the antioxidation strength of compounds in EAME was estimated by the rank of the known SAOx. If most of the SAOx were ranked high in the scoring system, then the scoring system is reliable to be applied to discover the antioxidants from plant extracts.

## Ultra-high-resolution mass spectra

**Fig. 8:**
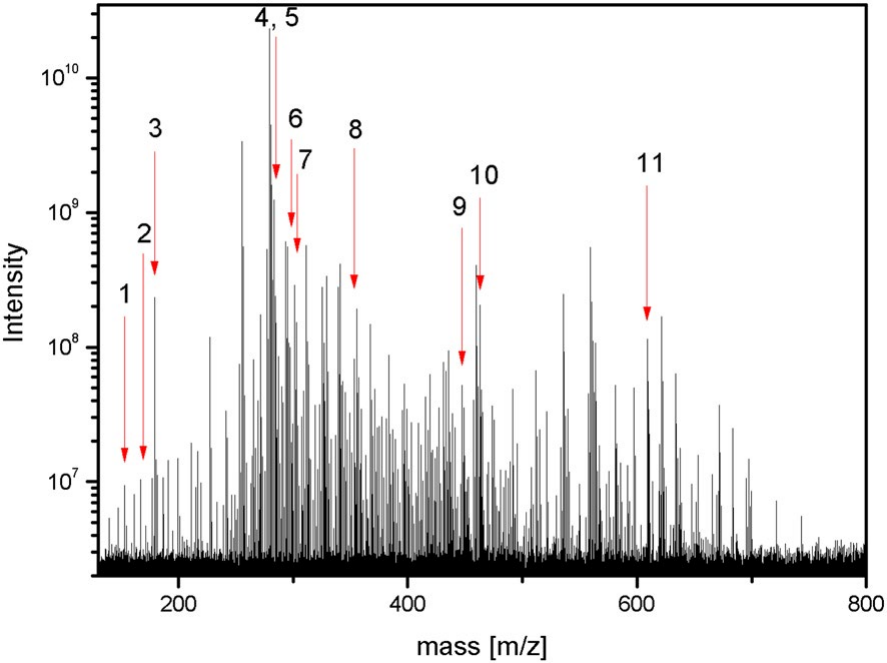
15 T FT-ICR mass spectrum of the EtOAc extract of mulberry fruit. A UHR mass spectrum of EAME was generated using a 15 T FT-ICR MS and all 11 SAOx were detected at high mass accuracy with measurement error < 0.05 mDa

A UHR mass spectrum of EAME was generated using a 15 T FT-ICR MS, as shown in Fig. 8. A total of 520,000 peaks were observed over a mass-to-charge ratio (m/z) range of 120–1200, with S/N > 4. We accumulated three blank mass spectra, where averaged blank spectra peaks were removed from the mass peak of each fraction to filter background peaks. The data were also reduced without loss of information using a de-isotoping procedure to reduce the computational burden. This was done by identifying ^13^C isotopic peaks and deleting up to three heavy carbon atoms in the corresponding chemical formula. Deisotoping is carried out as follows. The peaks were sorted by the intensity in the descending order. Then starting from the highest intensity peak, the existence of C^13^ or 2C^13^ isotope peaks was tested by comparing the calculated and observed mass values and those isotopic peaks were removed from the candidate list if existed. Scripts for calculating deisotoping and scoring were written in PERL programming language and are available from http://zemanet.net/scamp. A UHR mass profile of the compounds in EAEM was obtained and all 11 SAOx were detected at high mass accuracy (measurement error < 0.05 mDa), as shown in Table 2. Almost all SAOx chemical formulae can be directly determined with accurate mass values and isotopic distributions from UHR mass spectra. Some molecules do not show strong mass spectral intensities within the total extract mixture; however, if they are separated into 33 fractions then most peak intensities become stronger due to the collection of molecules with similar physical properties within the same chromatographic fraction. UHR mass spectra from all 33 fractions were acquired three times for each fraction using a 15-T FT-ICR MS and the average mass spectrum was used as a mass profile in SCAMP calculations.

## SCAMP scoring system

A mass spectrum can be divided into multiple m/z bins based on the resolution of the mass spectrometer used. For example, the m/z range from 120 to 1200 can be divided into 1081 bins with unit resolution. A mass profile of the jth m/z bin can be represented by the vector M, where M_j_ = {m_1j_, m_2j_, m_3j_, …, m_Nj_; N = fraction number}. In this equation, m_kj_ = the mass peak intensity of the jth m/z bin of fraction k, and N is the total number of fractions. If there is more than one peak within an m/z bin, their intensities are summed to assign m_kj_. In the same way, the activity profile vector is defined as A = {a_1_, a_2_, a_3_, …, a_N_}, where a_k_ is the activity of fraction k. The original SCAMP algorithm only considered the similarity between mass intensity and activity changes through all fractions. The first previous scoring method [5] summed the product of the normalized mass intensity vector (M_j_^0^ = {m_1j_^0^, m_2j_^0^, m_3j_^0^, …, m_Nj_^0^; N = fraction number}), and normalized activity vector (A^0^ = {a_1_^0^, a_2_^0^, a_3_^0^, …, a_N_^0^}) through all 33 fractions. The activity and mass profile vectors were normalized such that |A^o^| = 1 and |M_j_^o^| = 1 prior to the calculation of correlation coefficients. Only the relative changing trend was compared, where the total amount of active components was not considered.Scoring method 1:

$$ {\text{Score}}\;1 = C_{j} = \sum\limits_{K = 1}^{N} {a_{k}^{0} m_{kj}^{0} } $$

*j* = mass bin sequence number, *k* = fraction number, *C*_*j*_ = correlation coefficient, $a_{k}^{0}$ = normalized activity, $m_{kj}^{0}$ = normalized mass intensity of the *j*th mass peak in fraction *k.*

To reduce the noise peak, three blank mass spectra were averaged and removed from the mass spectrum of each fraction. ^13^C isotope peaks were removed from the peak list to reduce the calculation time using the mass measurement accuracy of 15 T FT-ICR MS. Initially, ~ 520,000 peaks with a mass bin size of 0.5 ppm were screened using de-isotoping and blank peak deletion (*C*_*j*_ > 33) to reduce calculation time, resulting in ~ 8000 peaks. Thus, the correlation coefficient *C*_*j*_ indicates the relative strength of antioxidant candidates.

Thus, intensity is not considered as a component of the correlation coefficient in scoring method 1. In real situations, if the concentration of a given component is below the detection limit of the mass spectrometer, then it would not be evident in the mass spectrum regardless of its activity. In addition, if the concentration of a given antioxidant is higher in certain fractions, then the antioxidative activities of those fractions would also be higher. The amount of a given antioxidant across multiple fractions can be calculated as the sum of peak intensities at a specific mass, in addition to the behavioral similarity index (correlation coefficient *C*_*j*_), which was used as a new factor to indicate the concentration dependence of the activity profile. To compensate for the effects of concentration on antioxidative components included in the extract, we summed antioxidant mass peak (*j*) intensities through all fractions (*k*) and multiplied this by the original *C*_*j*_, resulting in the second improved scoring method.

Scoring method 2:

$$ {\text{Score}}\;2 = C_{j} \times I $$

*C*_*j*_ = correlation coefficient, *I* = peak (*j*) intensities summation through all fractions (*k*).

**Fig. 9:**
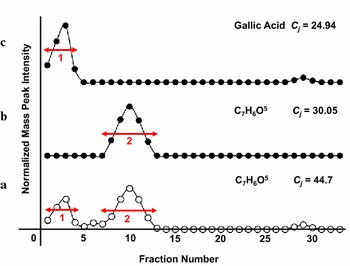
Grouping method and its influence on correlation coefficients. If some components have the same mass (169.014 u) but two different structures, as in (a), then those two components will present two separate chromatographic distributions within different fractions, described as regions 1 in (c), and 2 in (b). If the correlation coefficient *C*_*j*_ is calculated using scoring method 1 without a grouping process then Score 1 (44.7, (a)) will be much higher than the *C*_*j*_ of gallic acid (24.94, (c)) due to the different component in the same mass peak

The third scoring method clusters different components within the same mass peak, but having different fraction-centered chromatographic distributions within sequential fractions, as shown in Fig. 9. For example, if some components have the same mass (169.014 u) but two different structures, as in Fig. 9a, then those two components will present two separate chromatographic distributions within different fractions, described as regions 1 in Fig. 9c, and 2 in Fig. 9b. If the correlation coefficient *C*_*j*_ is calculated using scoring method 1 without a grouping process then Score 1 (44.7, Fig. 9a) will be much higher than the *C*_*j*_ of gallic acid (24.94, Fig. 9c) due to the different component in the same mass peak (which is usually one M_j_, but can represent several structural isomers). It is thus necessary to group the fractions included in different chromatographic distributions, and consider those separated groups as a new mass bin (jth m/z bin, M_j_) that is generated for different compounds. For these groupings, the mean peak intensity was first calculated using intensity values of fractions in each m/z. The algorithm then passed over data to find contiguous runs of values either less than or greater than the mean. Contiguous regions with intensity values greater than the mean became peaks, and the rest troughs. This result yielded a much lower *C*_*j*_ after the grouping process, as shown in Fig. 9b, c. The grouping strategy was adopted to improve the performance of SCAMP by avoiding overestimation of the correlation coefficient by providing additional scores for multiple components within the same mass peak, as in Fig. 9.

Scoring method 3:

$$ {\text{Score}}\;3 = \frac{{C_{j} \sigma^{2} I}}{{N_{f}^{2} }} $$

*C*_*j*_ = correlation coefficient, σ = standard deviation, *I* = intensity summation, *N*_f_ = number of fractions in the group.

A candidate list of antioxidants in EAEM was produced using scoring method 3. Five SAOx were determined quickly in the high-score regions of the candidate list, as shown in Additional file 1: Table S1. Normalized activities for each fraction are listed in the last line of the table, and all normalized mass peak intensities are listed for each mass bin for all 33 fractions. All data were sorted by Score 3, which avoids overestimation of the score by grouping multiple components in the same mass bin. Five SAOx were observed within the top 20, indicating that we can quickly enrich active antioxidants (Table 2).

**Table 2:** Identification of standard anti-oxidant compounds in the EtOAc extract of mulberry fruit using UHR 15-T FT-ICR mass spectrometry

#	Standard name	Formula	Calc. [M-H]^−^ [m/z]	Observed [M-H]^−^ [m/z]	Error [mDa]	S/N
1	Gentisic acid	C_7_H_6_O_4_	153.01933	153.01933	0.00	24
2	Gallic acid	C_7_H_6_O_5_	169.01425	169.01426	0.01	10.1
3	Caffeic acid	C_9_H_8_O_4_	179.03498	179.03498	0.00	18.5
4	Luteolin	C_15_H_10_O_6_	285.04046	285.04047	− 0.01	372.3
5	Kaempferol	C_15_H_10_O_6_	285.04046	285.04047	− 0.01	372.3
6	Quercetin	C_15_H_10_O_7_	301.03538	301.03538	0.00	710.1
7	Dihydroquercetin	C_15_H_12_O_7_	303.05103	303.05104	− 0.01	370.6
8	Chlorogenic acid	C_16_H_18_O_9_	353.08781	353.08780	0.01	191.4
9	Quercitrin	C_21_H_20_O_11_	447.09329	447.09326	0.03	53.9
10	Quercetin-3-*o*-glucoside	C_21_H_20_O_12_	463.08820	463.08820	0.00	467.9
11	Rutin	C_27_H_30_O_16_	609.14611	609.14610	0.01	245.5

A UHR mass profile of the compounds in EAEM was obtained and all 11 SAOx were detected at high mass accuracy with measurement error < 0.05 mDa

## Scoring algorithm estimation

**Fig. 10:**
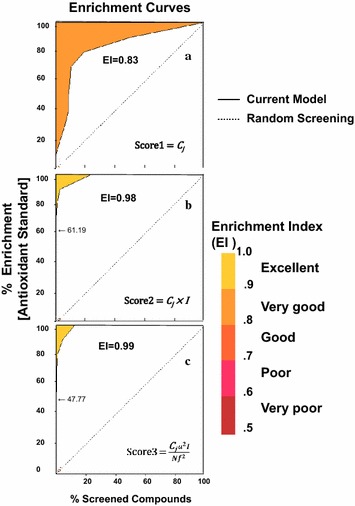
Active compound enrichment curves by different scoring methods. **a** The antioxidative compound enrichment curve (EI = 0.83) of the scoring method 1 was obtained for correlation coefficients *C*_*j*_ between normalized activity and mass profiles as previous SCAMP methods. **b** The antioxidative compound enrichment curve (EI = 0.98) of the scoring method 2. To compensate for the effects of concentration on antioxidative components included in the extract, we summed antioxidant mass peak intensities through all fractions (*k*) and multiplied this by the original *C*_*j*_, resulting in the second improved scoring method. **c** The enrichment curve (EI = 0.99) for the third scoring method. If some components have the same mass but different structures, then those two components will present two separate chromatographic distributions and within different fractions. The grouping strategy was adopted to improve the performance of SCAMP by avoiding overestimation of the correlation coefficient by providing additional scores for multiple components within the same mass peak

In this study, we suggest new scoring algorithms that introduce new influencing factors into the scoring system to improve the efficiency of active compound enrichment. A new concentration dependence factor for the activity profile was introduced in scoring method 2, while the third method clusters different components in the same mass peak, as in Fig. 9. SCAMP methods were validated by their enrichment factors [21–25]. Enrichment factor is defined as the ratio of the observed fraction of active compounds in the top few percent of screening to a random distribution [22]. It is widely used in virtual screening of chemical compounds for drug discovery for assessing the quality of screening protocol. Enrichment curves were obtained by plotting percent of actives found against percent of database screened and enrichment factors were calculated as the area under curve (AUC), using a plot_enrich program which is available from http://zemanet.net/plot_enrich/. Using those two new algorithms, enrichment curves were calculated to analyze the ability of different scoring methods to identify active compounds. These curves show how the fraction of active compound recovered varies with the percent compound screened. We also calculated the area under curve (AUC) to compare the scoring quantitatively. The AUC ranged from 0 to 1, where an AUC of 1.0 indicates an ideal scoring method and an AUC of 0.5 indicates a random selection distribution. Figure 10a shows enrichment curves obtained for correlation coefficients *C*_*j*_ between normalized activity and mass profiles as per previous SCAMP methods. The mass peak intensities were considered to be a major influencing factor in scoring method 2 (Score 2), as shown in Fig. 10b, in which de-isotoping and blank peak deletion were applied (where *C*_*j*_ values less than 30 were removed). Figure 10c shows enrichment curves for the third scoring method (Score 3) where the grouping of fractions was applied. AUC values calculated for the original *C*_*j*_ value were 0.83, while those for methods 2 and 3 were 0.98 and 0.99, respectively. Curves obtained for the correlation coefficients *C*_*j*_ (as in the previous SCAMP) deviate from the random screening line and AUC, meaning that this method detects active compounds reasonably well. The second method yielded improved enrichment plots, as confirmed by an AUC value of 0.98. Thus, the concentration effect cannot be ignored for SCAMP calculations. Finally, a clustering strategy can improve the performance of SCAMP by considering structural isomers having the same mass. When compared to the previous method 1, the methods 2 and 3 both greatly improved performance by the concentration factor, as confirmed by AUC values of 0.98 and 0.99, respectively. Overall, enrichment curves and AUC values calculated for methods 1 and 2 demonstrate that method 2 performs better than method 1; however, both are better than using the correlation coefficient *C*_*j*_.

**Fig. 11:**
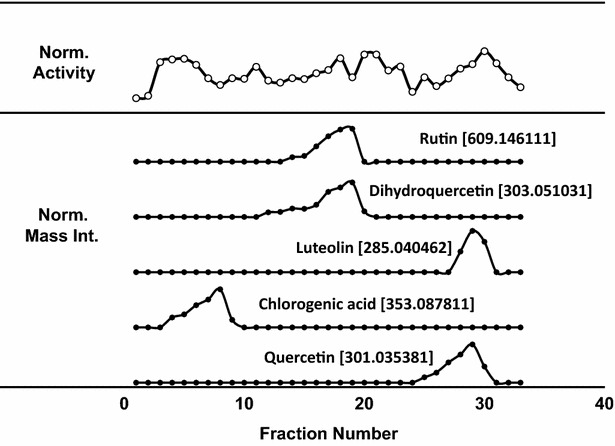
Antioxidation activity and mass intensities of standard antioxidants profiles. Five strong standard antioxidants in the high-score candidate region validate the efficiency of the scoring method to enrich the antioxidants from the components in complicated plant extract mixture. The mass peak intensities of five strong standard antioxidants showed the strong correlation with complicated activity profiles through the all fractions. Due to a relatively polar sugar ring, chlorogenic acid was eluted at the very beginning and luteolin and quercetin were eluted in the later fractions as similar eluting sequence in UPLC/MS

**Fig. 12:**
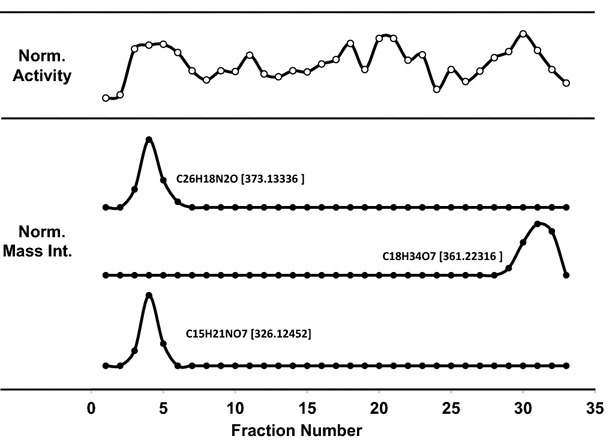
Antioxidation activity and mass intensities of three new candidates. Three new compounds showed the close correlation with activity profiles and probably the very early eluting fractions and the last fractions will be the good starting fractions to find the new oxidative molecules from mulberry fruits

Finally, a top 20 anti-oxidant candidate list was generated by sorting the mass peak list according to Score 3. The corresponding formulae are given in Table 3. Five strong standard antioxidants [12, 13, 15] and two additional antioxidants previously found in mulberry fruits [26, 27] in the high-score candidate region validate the efficiency of the scoring method to enrich the antioxidants from the components in complicated plant extract mixture. The mass peak intensities of five strong standard antioxidants showed the strong correlation with complicated activity profiles through the all fractions as shown in Fig. 11. Due to a relatively polar sugar ring, chlorogenic acid (14.2 ng) was eluted at the very beginning and dihydoxy quercetin (46.2 ng), rutin (154.0 ng), quercetin (71.7 ng) and luteolin (3.5 ng) were eluted in the later fractions as similar eluting sequence in UPLC/MS in Fig. 3 and also observed in different working groups as shown in Additional file 2: Table S2. From a survey of natural product databases [28] and reference literatures, six of these antioxidants are found in other plants [29–31] and most of the antioxidants have structures corresponding to flavonoids. Four known compounds was listed in the top 20 list and never published as an antioxidant before. So it can be a new antioxidant candidates. And three new compounds showed the close correlation with activity profiles as shown in Fig. 12. Probably the very early eluting fractions and the last fractions will be the good starting fractions to find the new oxidative molecules from mulberry fruits.

**Table 3:** Discovered antioxidant candidates using the enhanced scoring system

#	Antioxidant compounds	Formular	Molecular weight	Monoisotopic mass m/z of [M-H]^−^	MS/MS fragment ion peaks	Score 3	Antioxidant	Plant	Database ID	References
1	(R)-Fulvic acid	C_14_H_12_O_8_	308.242	307.04595	171.10(28) 209.11(17) 291.19(18) 307.19(100)	19,604	–	–	51,382,658	–
2	Corchori fatty acid F	C_18_H_32_O5	328.444	327.21770	171.10(42) 211.13(38) 229.14(31) 327.22(100)	15,916	–	*Corchorus olitorius* L.	44,559,173	[32]
3	***Quercetin***	C_15_H_10_O_7_	302.238	301.03538	175.04(19) 151.00(32) 149.02(10) 107.01(13)	9383	***SAOx***	***Morus alba*** **L.**	5,280,343	[13]
4	***Methyl syringin***	C_18_H_26_O_9_	386.397	385.15040	223.10(100) 385.15(55)	8167	–	***Morus alba*** **L.** *Styrax japonica* S.	FDB019191	[33]
5	***Chlorogenic acid***	C_16_H_18_O_9_	354.311	353.08781	191.05(100)	7026	***SAOx***	***Morus alba*** **L.**	179,4427	[12]
6	New compound 1	C_15_H_21_NO_7_	327.329	326.12452	N.A.	6918	***N1***	–	–	–
7	5-O-Caffeoylshikimic acid	C_16_H_16_O_8_	336.296	335.07725	N.A.	5841	***AOx1***	Phoenix dactylifera	5,281,762	[30]
8	***Naringenin***	C_15_H_12_O_5_	272.252	271.06119	119.05(44) 151.00(100) 177.02(19) 271.06(66)	5326	***O1***	***Morus alba*** **L.**	439,246	[26]
9	2-formyl-5-(hydroxymethyl)-1H-pyrrole-1-butanoic acid	C_10_H_13_NO_4_	211.214	210.07725	124.04(99) 210.07(100)	5261	***O2***	***Morus alba*** **L.**	–	[27]
10	Butyl buty ryllactate	C_11_H_20_O_4_	216.274	215.12890	179.05(17) 215.03(100)	5150	–	*Fragaria ananassa* Duch.	24,114	–
11	Caohuoside C	C_27_H_30_O_11_	530.526	529.17149	N.A.	4993	***AOx2***	*Epimedium koreanum*	44,259,411	[34]
12	Manniflavanone	C_30_H_22_O_13_	590.493	589.09881	N.A.	4864	***AOx3***	*Garcinia buchananii*	198,549	[35]
13	Crocusatin H	C_12_H_20_O_4_	228.288	227.12893	138.01(12) 209.19(30) 227.20(100)	4478	***AOx4***	*Crocus sativus*	637,013	[31]
14	***Luteolin***	C_15_H_10_O_6_	286.239	285.04046	133.02(100) 149.02(10) 151.00(32) 199.04(13)	4434	***SAOx***	***Morus alba*** **L.**	5,280,445	[15]
15	***Dihydroquercetin***	C_15_H_12_O_7_	304.254	303.05103	125.02(20) 177.02(18) 285.04(100)	4361	***SAOx***	***Morus alba*** **L.**	439,533	[15]
16	Luteolin 7-O-glucuronide	C_21_H_18_O_12_	462.363	461.07256	301.04 (10) 461.07(100)	4199	***AOx5***	*Acanthus hirsutus*	5,280,601	
17	***Rutin***	C_27_H_30_O_16_	610.521	609.14611	301.03(75) 300.02(100) 302.04(14)	4130	***SAOx***	***Morus alba*** **L.**	5,280,805	[13]
18	Dalspinin	C_17_H_12_O_7_	328.276	327.05104	255.23(20) 327.29(100)	4012	***AOx6***	*Dalbergia spinosa* Roxb	5,323,563	[29]
19	New compound 2	C_18_H_34_O_7_	362.463	361.22316	N.A.	3888	***N2***	–	–	–
20	New compound 3	C_26_H_18_N_2_O	374.141	373.13336	191.03(38) 327.05(25) 373.09(100)	3829	***N3***	–	–	–

The top 20 anti-oxidant candidate list of mulberry fruits was generated by sorting the mass peak list according to Score 3. The corresponding formulae are given by UHR FT-ICR MS. Five strong standard antioxidants (**SAOx**) were included in the list and two additional antioxidants (**O1**, **O2**) were found from the mulberry (*Morus alba* L.) fruit extract. Three unknown strong antioxidant candidates (**N1**, **N2**, and **N3**) were listed. From a survey of natural product databases [28] and reference literatures, six of these antioxidants (**AOx1 ~AOx6)** are found in other plants [29–31, 34, 35] and most of the antioxidants have structures corresponding to flavonoids. Four known compounds was never published as an antioxidant before. So it can be a new antioxidant candidates. And three new compounds (**N1**, **N2**, and **N3**) showed the close correlation with activity profiles. Probably the very early eluting fractions and the last fractions will be the good starting fractions to find the novel oxidative molecules from mulberry fruits

## Conclusions

Antioxidant-rich mulberry fruit extract was separated into 33 fractions and analyzed using high-resolution LC/MS, LC/MSMS, and UHR FT-ICR MS. The performance of our antioxidative component discovery method was validated by measuring the antioxidation strength of all 33 fractions using the correlation between mass and activity profiles from all chromatographic fractions. Proprietary scoring algorithms were used to enrich active components and detect several strong antioxidants within the mulberry extract. The AUC was measured to compare the scoring methods quantitatively. Two new scoring systems were compared to previous scoring parameters, where the calculated AUCs of both scoring systems (1 = 0.98 and 2 = 0.99) are higher than using a previous correlation coefficient *C*_*j*_ (0.89). Using an improved SCAMP enrichment system, thirteen unknown antioxidants were ranked higher than known standard antioxidants candidates in addition to known antioxidants, methyl syringin and naringenin (3.5 ng). The mass and retention time-targeted purification of unknown antioxidant candidates was shown to significantly reduce the purification time and labor involved in activity screenings.

## Additional files

**Additional file 1: Table S1.** Anti-oxidant discovery scoring data. Normalized activities for each fraction and all normalized mass peak intensities for each mass bin for all 33 fractions are listed and sorted by the values of Score 3. Grouping information also is provided by fraction number of center position and total number of fractions in each group.

**Additional file 2: Table S2.** Levels of antioxidants in mulberry fruits published. Levels of anti-oxidant compounds included in mulberry fruits were measured with LC/MS by comparing the UV absorption chromatograms and selected ion chromatograms of SAOx and EAEM. Other observed levels are also compared.

## Abbreviations

EAEM: mulberry ethyl acetate extract, ethyl acetate extract of mulberry fruits
SAOx: standard antioxidants
ESI: electrospray ionization
FT-ICR MS: Fourier transform ion cyclotron resonance mass spectrometry
HPLC: high-performance liquid chromatography
HR MS: high-resolution mass spectrometry
LC: liquid chromatography
MS/MS: tandem mass spectrometry
S/N: signal-to-noise ratio
UHR: ultra-high resolution
