# Supplementary material for: High-throughput detection of antioxidants in mulberry fruit using correlations between high-resolution mass and activity profiles of chromatographic fractions
Source: Plant Methods. 2017 Dec 6;13:108. doi: 10.1186/s13007-017-0258-3 (PMC5718003; doi:10.1186/s13007-017-0258-3)
Supplement: Supplementary file 1 — Additional file 1: Table S1. Anti-oxidant discovery scoring data. Normalized activities for each fraction and all normalized mass peak intensities for each mass bin for all 33 fractions are listed and sorted by the values of Score 3. Grouping information also is provided by fraction number of center position and total number of fractions in each group. [file 13007_2017_258_MOESM1_ESM.pptx]

## Slide 1
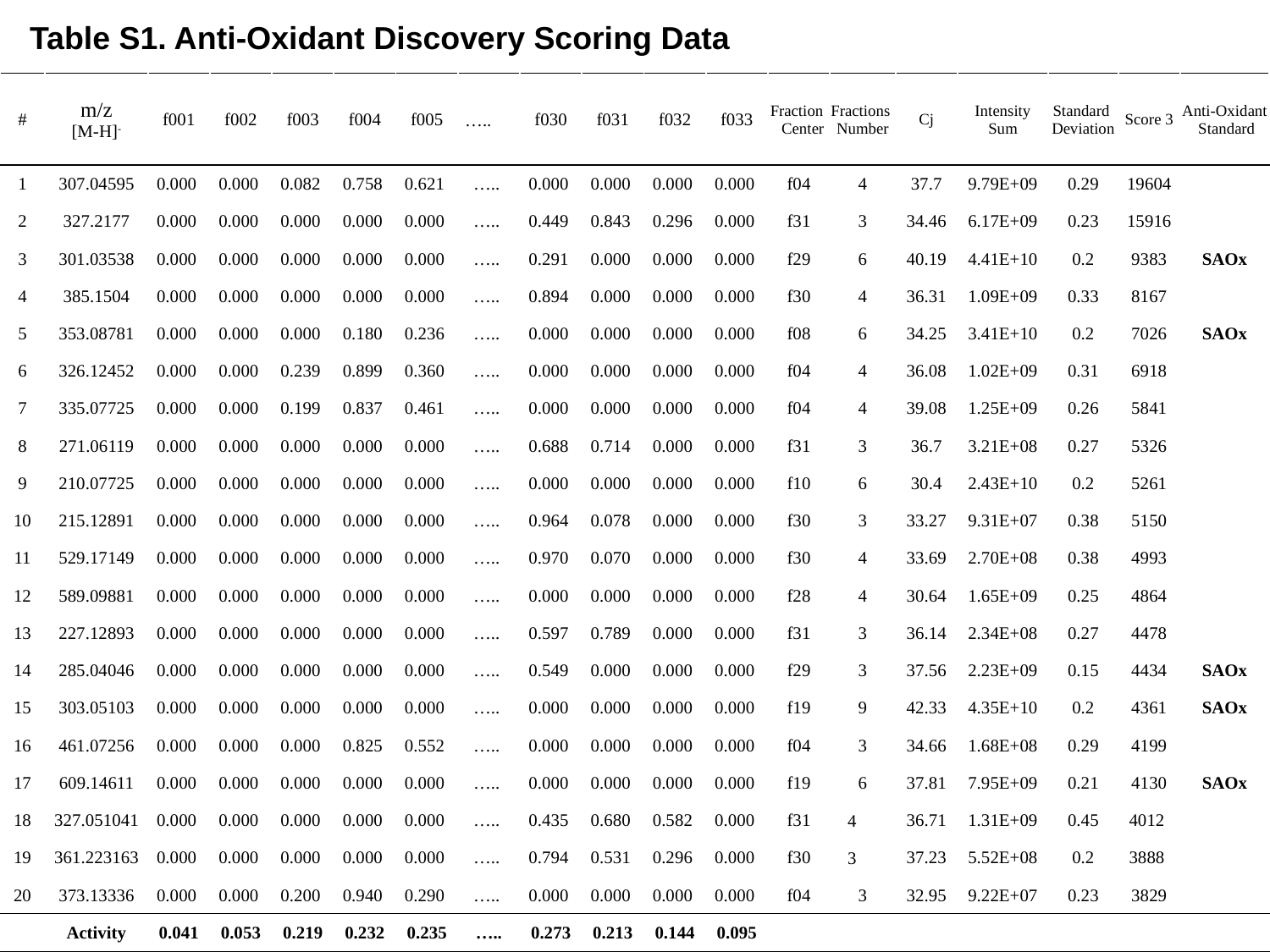

Table S1. Anti-Oxidant Discovery Scoring Data
| # | m/z [M-H]- | f001 | f002 | f003 | f004 | f005 | ….. | f030 | f031 | f032 | f033 | Fraction Center | Fractions Number | Cj | Intensity Sum | Standard Deviation | Score 3 | Anti-Oxidant Standard |
| --- | --- | --- | --- | --- | --- | --- | --- | --- | --- | --- | --- | --- | --- | --- | --- | --- | --- | --- |
| 1 | 307.04595 | 0.000 | 0.000 | 0.082 | 0.758 | 0.621 | ….. | 0.000 | 0.000 | 0.000 | 0.000 | f04 | 4 | 37.7 | 9.79E+09 | 0.29 | 19604 | |
| 2 | 327.2177 | 0.000 | 0.000 | 0.000 | 0.000 | 0.000 | ….. | 0.449 | 0.843 | 0.296 | 0.000 | f31 | 3 | 34.46 | 6.17E+09 | 0.23 | 15916 | |
| 3 | 301.03538 | 0.000 | 0.000 | 0.000 | 0.000 | 0.000 | ….. | 0.291 | 0.000 | 0.000 | 0.000 | f29 | 6 | 40.19 | 4.41E+10 | 0.2 | 9383 | SAOx |
| 4 | 385.1504 | 0.000 | 0.000 | 0.000 | 0.000 | 0.000 | ….. | 0.894 | 0.000 | 0.000 | 0.000 | f30 | 4 | 36.31 | 1.09E+09 | 0.33 | 8167 | |
| 5 | 353.08781 | 0.000 | 0.000 | 0.000 | 0.180 | 0.236 | ….. | 0.000 | 0.000 | 0.000 | 0.000 | f08 | 6 | 34.25 | 3.41E+10 | 0.2 | 7026 | SAOx |
| 6 | 326.12452 | 0.000 | 0.000 | 0.239 | 0.899 | 0.360 | ….. | 0.000 | 0.000 | 0.000 | 0.000 | f04 | 4 | 36.08 | 1.02E+09 | 0.31 | 6918 | |
| 7 | 335.07725 | 0.000 | 0.000 | 0.199 | 0.837 | 0.461 | ….. | 0.000 | 0.000 | 0.000 | 0.000 | f04 | 4 | 39.08 | 1.25E+09 | 0.26 | 5841 | |
| 8 | 271.06119 | 0.000 | 0.000 | 0.000 | 0.000 | 0.000 | ….. | 0.688 | 0.714 | 0.000 | 0.000 | f31 | 3 | 36.7 | 3.21E+08 | 0.27 | 5326 | |
| 9 | 210.07725 | 0.000 | 0.000 | 0.000 | 0.000 | 0.000 | ….. | 0.000 | 0.000 | 0.000 | 0.000 | f10 | 6 | 30.4 | 2.43E+10 | 0.2 | 5261 | |
| 10 | 215.12891 | 0.000 | 0.000 | 0.000 | 0.000 | 0.000 | ….. | 0.964 | 0.078 | 0.000 | 0.000 | f30 | 3 | 33.27 | 9.31E+07 | 0.38 | 5150 | |
| 11 | 529.17149 | 0.000 | 0.000 | 0.000 | 0.000 | 0.000 | ….. | 0.970 | 0.070 | 0.000 | 0.000 | f30 | 4 | 33.69 | 2.70E+08 | 0.38 | 4993 | |
| 12 | 589.09881 | 0.000 | 0.000 | 0.000 | 0.000 | 0.000 | ….. | 0.000 | 0.000 | 0.000 | 0.000 | f28 | 4 | 30.64 | 1.65E+09 | 0.25 | 4864 | |
| 13 | 227.12893 | 0.000 | 0.000 | 0.000 | 0.000 | 0.000 | ….. | 0.597 | 0.789 | 0.000 | 0.000 | f31 | 3 | 36.14 | 2.34E+08 | 0.27 | 4478 | |
| 14 | 285.04046 | 0.000 | 0.000 | 0.000 | 0.000 | 0.000 | ….. | 0.549 | 0.000 | 0.000 | 0.000 | f29 | 3 | 37.56 | 2.23E+09 | 0.15 | 4434 | SAOx |
| 15 | 303.05103 | 0.000 | 0.000 | 0.000 | 0.000 | 0.000 | ….. | 0.000 | 0.000 | 0.000 | 0.000 | f19 | 9 | 42.33 | 4.35E+10 | 0.2 | 4361 | SAOx |
| 16 | 461.07256 | 0.000 | 0.000 | 0.000 | 0.825 | 0.552 | ….. | 0.000 | 0.000 | 0.000 | 0.000 | f04 | 3 | 34.66 | 1.68E+08 | 0.29 | 4199 | |
| 17 | 609.14611 | 0.000 | 0.000 | 0.000 | 0.000 | 0.000 | ….. | 0.000 | 0.000 | 0.000 | 0.000 | f19 | 6 | 37.81 | 7.95E+09 | 0.21 | 4130 | SAOx |
| 18 | 327.051041 | 0.000 | 0.000 | 0.000 | 0.000 | 0.000 | ….. | 0.435 | 0.680 | 0.582 | 0.000 | f31 | 4 | 36.71 | 1.31E+09 | 0.45 | 4012 | |
| 19 | 361.223163 | 0.000 | 0.000 | 0.000 | 0.000 | 0.000 | ….. | 0.794 | 0.531 | 0.296 | 0.000 | f30 | 3 | 37.23 | 5.52E+08 | 0.2 | 3888 | |
| 20 | 373.13336 | 0.000 | 0.000 | 0.200 | 0.940 | 0.290 | ….. | 0.000 | 0.000 | 0.000 | 0.000 | f04 | 3 | 32.95 | 9.22E+07 | 0.23 | 3829 | |
| | Activity | 0.041 | 0.053 | 0.219 | 0.232 | 0.235 | ….. | 0.273 | 0.213 | 0.144 | 0.095 | | | | | | | |
