# Supplementary material for: High-throughput detection of antioxidants in mulberry fruit using correlations between high-resolution mass and activity profiles of chromatographic fractions
Source: Plant Methods. 2017 Dec 6;13:108. doi: 10.1186/s13007-017-0258-3 (PMC5718003; doi:10.1186/s13007-017-0258-3)
Supplement: Supplementary file 2 — Additional file 2: Table S2. Levels of antioxidants in mulberry fruits published. Levels of anti-oxidant compounds included in mulberry fruits were measured with LC/MS by comparing the UV absorption chromatograms and selected ion chromatograms of SAOx and EAEM. Other observed levels are also compared. [file 13007_2017_258_MOESM2_ESM.pptx]

## Slide 1
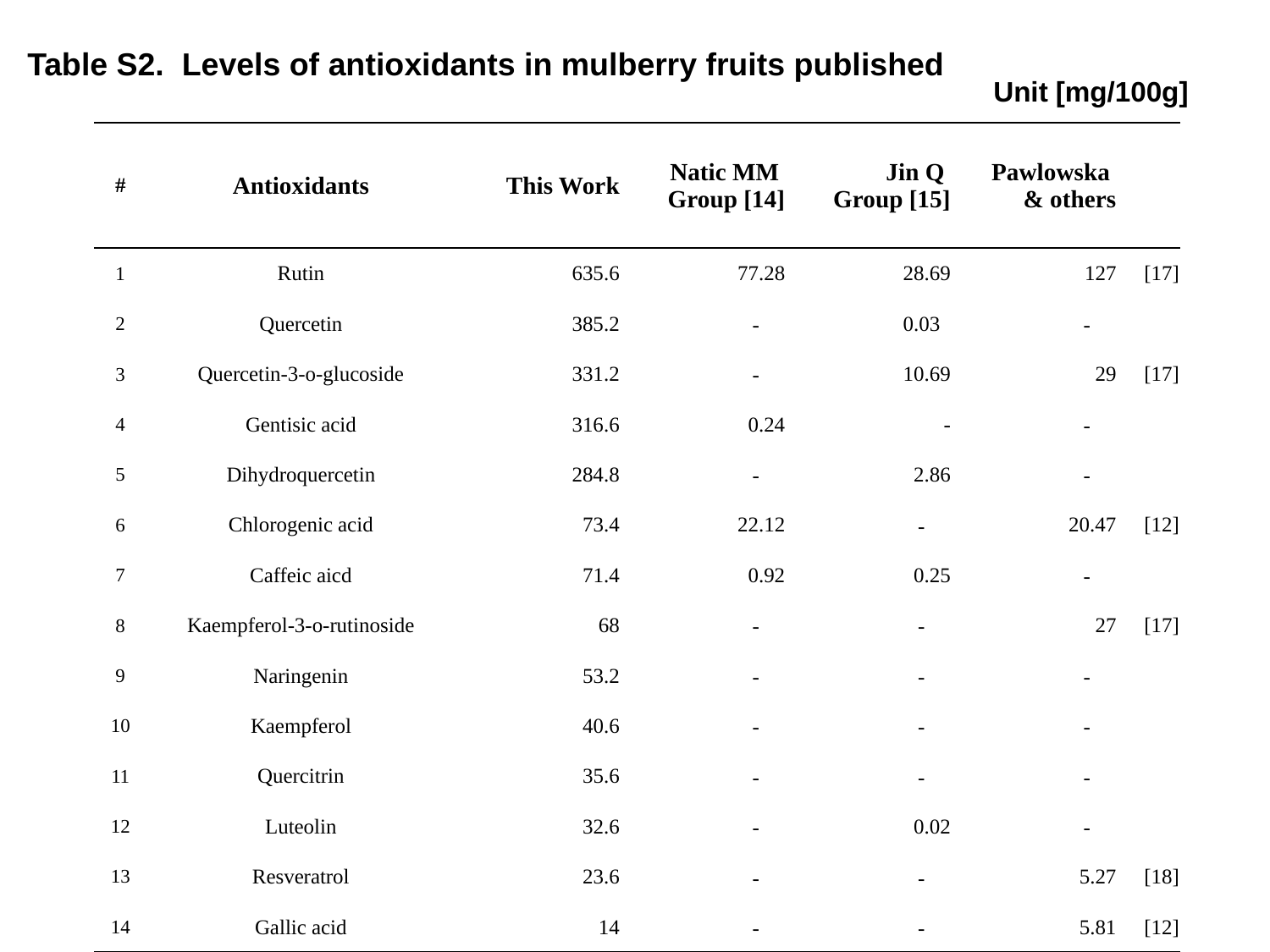

Table S2. Levels of antioxidants in mulberry fruits published
Unit [mg/100g]
| # | Antioxidants | This Work | Natic MM Group [14] | Jin Q Group [15] | Pawlowska & others | |
| --- | --- | --- | --- | --- | --- | --- |
| 1 | Rutin | 635.6 | 77.28 | 28.69 | 127 | [17] |
| 2 | Quercetin | 385.2 | - | 0.03 | - | |
| 3 | Quercetin-3-o-glucoside | 331.2 | - | 10.69 | 29 | [17] |
| 4 | Gentisic acid | 316.6 | 0.24 | - | - | |
| 5 | Dihydroquercetin | 284.8 | - | 2.86 | - | |
| 6 | Chlorogenic acid | 73.4 | 22.12 | - | 20.47 | [12] |
| 7 | Caffeic aicd | 71.4 | 0.92 | 0.25 | - | |
| 8 | Kaempferol-3-o-rutinoside | 68 | - | - | 27 | [17] |
| 9 | Naringenin | 53.2 | - | - | - | |
| 10 | Kaempferol | 40.6 | - | - | - | |
| 11 | Quercitrin | 35.6 | - | - | - | |
| 12 | Luteolin | 32.6 | - | 0.02 | - | |
| 13 | Resveratrol | 23.6 | - | - | 5.27 | [18] |
| 14 | Gallic acid | 14 | - | - | 5.81 | [12] |
